# Evolution of blood pressure in children with congenital and acquired solitary functioning kidney

**DOI:** 10.1186/s13052-017-0359-7

**Published:** 2017-04-27

**Authors:** Riccardo Lubrano, Isotta Gentile, Raffaele Falsaperla, Giovanna Vitaliti, Alessia Marcellino, Marco Elli

**Affiliations:** 1 Pediatric Department, Pediatric Nephrology Unit, Sapienza University of Rome, Viale Regina Elena 324, 00161 Rome, Italy; 2 General Pediatrics and Pediatric Acute and Emergency Unit, Policlinico-Vittorio-Emanuele University Hospital, Catania, Italy; 3 DiBiC-Biomedical and Clinic Science Department, “Luigi Sacco” - University of Milan, Milan, Italy

**Keywords:** Solitary functioning kidney, Glomerular filtration rate, Renal scar, Hypertension, Pre-hypertension, Blood pressure

## Abstract

**Background:**

It is not yet clear if blood pressure and renal function changes evolve differently in children with a congenital or acquired solitary functioning kidney. This study aims to assess if there are any differences between these two types of solitary kidney patients.

**Methods:**

Current research is a retrospective study assessing the evolution of glomerular filtration rate, proteinuria, and blood pressure in clinical records of 55 children with a solitary functioning kidney (37 congenital and 18 acquired). We used the medical records of children who had been assisted, in our unit of pediatric nephrology, for a period of 14 years (168 months), from the time of diagnosis, between January/1997 and December/2015.

**Results:**

During the study period, glomerular filtration rate (T0 128.89 ± 32.24 vs T14 118.51 ± 34.45 ml/min/1.73 m^2^, p NS) and proteinuria (T0 85.14 ± 83.13 vs T14 159.03 ± 234.66 mg/m^2^/die, p NS) demonstrated no significant change. However, after 14 years of follow-up 76.4% of patients had increased levels of arterial hypertension with values over the 90th percentile for gender, age, and height. Specifically, children with an acquired solitary functioning kidney mainly developed hypertension [T0 2/17 (12%) vs T14 9/17 (52.9%) *p* < 0.025], whereas children with a congenital solitary functioning kidney mainly developed pre-hypertension [T0 3/38 (7.9%) vs T14 17/38 (44.7%) *p* < 0.0005].

**Conclusions:**

The renal function of children with solitary functioning kidneys remains stable during a follow-up of 14 years. However, these children should be carefully monitored for their tendency to develop arterial blood pressure greater than the 90th percentile for gender, age, and height.

## Background

In the 1980s, Brenner et al. [1–3] theorized, based on observational animals models, that the compensatory hypertrophy of the solitary functioning kidney (SFK) would cause hyperfiltration in the residual nephrons; leading to proteinuria and arterial hypertension and finally glomerulosclerosis and End Stage Renal Disease (ESRD) would happen. Human studies in SFK patients reported conflicting results. Siomou et al. [4] reported an increase of blood pressure Z-score values; while renal function and microalbumin urinary excretion were normal among congenital SFK children compared to control group. Contradictory, Dursun et al. [5] found a reduced renal function accompanied with normal blood pressure. While Shirzai et al. [6] found increased levels of 24-h urinary microalbumin only in children suffering from SFK with longer than 5-year duration since onset. On the other hand, Seeman et al. [7] reported blood pressure increase and reduced renal function in SFK children with abnormalities of their residual kidney.

Therefore, Siomou [4] highlighted the importance of increased serial controls in case of glomerular hyperfiltration signs; whereas Shirzai [6] supported clinical controls for the possible onset of microalbuminuria, only for subjects suffering from SFK for more than 5 years. Finally, Seeman [7] recommended strict clinical observation only for children with SKF and renal scarring.

Present study conducted a retrospective approach in a population of SFK children, in order to assess changes in renal function (glomerular filtration rate and proteinuria) and arterial blood pressure according to the congenital or acquired nature of the diseases. Patients were followed over a 168-month observation period.

## Methods

### Study population

This is a retrospective study based on the clinical records of pediatric patients with congenital or acquired SFK, with an observation time of 14 years (168 months). Current study aimed to assess if the nature of SFK (congenital vs. acquired) has a different influence on blood pressure and renal function.

### Inclusion and exclusion criteria

We enclosed all children referred to our center with a diagnosis of SFK that were aged between 3 months and 18 years at diagnosis; and followed them at our day hospital for 14 years. SFK subjects were divided into two groups: congenital SFK (cSFK) and acquired SFK (aSFK).

The cSFK group included patients with one kidney found absent or significantly smaller at ultrasound, with no detectable renal function or glomerular filtration rate (GFR) <10 ml/min/1.73 m^2^ at Tc-99 m DTPA renal scintigraphy, and no history of surgical nephrectomy.

The aSFK group included SFK patients with complete absence of one kidney due to previous mono-lateral total nephrectomy due to trauma.

Exclusion criteria included previous treatment with drugs potentially affecting renal function, documented orthostatic proteinuria, congenital anomalies of the urinary tract and SFK due to previous nephrectomy for causes other than trauma.

### Protocol of the study

For each patient, glomerular filtration rate, proteinuria, and blood pressure at the time of first admission (T0) and at 14 years (T14) were extrapolated from the records stored in our center for pediatric nephrology.

1. For the evaluation of renal function
  We measured proteinuria in 24-h urine collections as described previously [8]. In case of lack of voluntary control of micturition, we collected the urine for 8 h in daytime via urinary collection bags connected to a binder (U-Bag® Urine Specimen Collector).
  We used the Tc-99 m DTPA renal scintigraphy to measure the GFR of the two kidneys separately [9] and Tc-99 m DMSA renal scintigraphy to detect the presence of renal scars [10]
  As normal values, we assumed: GFR >90 ml/min/1.73 m^2^ [11, 12] and proteinuria <100 mg/m^2^/24 h [13].
2. Measurement, evaluation, and classification of blood pressure:
  In children <120 cm in height we only took blood pressure manually with a mercury sphygmomanometer (clinical blood pressure). In children ≥120 cm in height we added a 24-h ambulatory blood pressure monitoring (ABPM) applying the technical modalities described previously [14]. For all blood pressure measures we used a cuff appropriate to the size of the child’s upper arm according to the Fourth Report [15]. To classify the blood pressure status of the children we used centiles and the classification schemes of the following guidelines :
  In children between 0 and 18 years:
    Clinical blood pressure classification:
      In children less than 1 year, the tables for diagnosis of neonatal hypertension were used [16]
      In children between 1 and 18 years, we used “the Fourth Report on the Diagnosis, Evaluation and Treatment of High Blood Pressure in Children and Adolescents” [15]. as it separates 1) normal blood pressure, 2) hypertension, and 3) prehypertension.
    ABPM classification:
      The updated recommendations for the standard assessment of ABPM in children and adolescent were applied [17] which combines clinical blood pressure and ABPM value
      as it separates 1) normal blood pressure, 2) hypertension, and 3) prehypertension, 4) masked hypertension, and 5) with white coat hypertension
  In patients older than 18 years:
    The guidelines set by the European Society of Cardiology for the assessment of clinical blood pressure and ABPM were followed [18].
    as it separates adults with In 1) normal blood pressure, 2) hypertension, and 3) prehypertension, 4) masked hypertension, and 5) with white coat hypertension

### Ethical considerations

The study protocol conformed to the ethical guidelines of the 1975 Declaration of Helsinki as revised in 2000 [19] and was approved by the ethic committee of our institutions.

### Statistical analysis

For statistical evaluation, we used a dedicated software: JMP (produce of SAS Institute Inc. Cary, NC 27513–2414, USA) e GraphPad 5.0 (La Jolla, CA, U.S.A.). We expressed the qualitative variables as percent and the quantitative as mean ± standard deviation. We analyzed the differences between the groups using the chi-square (*χ* ^2^) test for data expressed as percent. For the data expressed as mean ± standard deviation we tested the approximation to normal of the distribution of the population by Kolmogorov-Smirnov One-Sample Test and statistics for kurtosis and symmetry. As results were asymmetrically distributed, non-parametric tests were used (Wilcoxon test).

We considered the differences with *p* < 0.05 significant.

## Results

### Characteristics of the study population

We examined the clinical records of 61 patients (44 males and 17 females) during 14 years after diagnosis at our center. Among them, 6 patients were excluded. Two of them had nephrectomy secondary to Wilms tumor, and four were affected by vesicoureteral reflux. Among remaining 55 patients (40 males and 15 females), 38 patients had congenital SFK and 17 of them experienced acquired form.

The mean age at diagnosis was significantly lower (*p* < 0.001) in the cSFK group (6.24 ± 3.55 months) compared to the aSFK group (95.11 ± 33.12 months). Causes of cSFK included unilateral renal agenesis in 21, congenital aplasia/hypoplasia in 8, and multicystic kidney disease in 9 children.

### Renal function

In Table 1 we report mean values of GFR and proteinuria in total population, and their mean values in each of the two subgroups (aSFK and cSFK) at T0 and T14. No significant differences between these groups were detected.

**Table 1:** GFR and proteinuria at time of diagnosis (T0) and after 14 years follow-up (T14)

	GFR ml/min/1.73 m^2			Proteinuria mg/m^2/die		
	T0	T14	*p*<	T0	T14	*p*<
SFK	128.89 ± 32.24	118.51 ± 34.45	NS	85.14 ± 83.13	159.03 ± 234.66	NS
aSFK	109.41 ± 33.71	100.72 ± 33.49	NS	61.35 ± 17.15	140.52 ± 122.26	NS
cSFK	126.60 ± 34.98	103.76 ± 46.49	NS	106.30 ± 111.78	147.15 ± 247.46	NS

*SFK* all solitary functioning kidney patients; *aSFK* acquired type; cSFK: congenital type

*GFR and Proteinuria at T0 and T14*: aSFK vs cSFK, SFK vs aSFK, SFK vs cSFK: p NS

The number of subjects with renal scar was similar at T0 in both groups (cSFK 10/38 vs aSFK 4/17 p NS); while it was significantly increased at T14 in subjects with cSFK (T0 10/38 vs T14 24/38 *p* < 0.003). This value remained stable in aSFK patients during the course of follow-up (T0 4/17 vs T14 5/17 p NS).

### Hypertension

Table 2 illustrates the partition of SFK patients congenital and acquired in normotensive, prehypertensive, and hypertensive at T0 and T14.

**Table 2:** Blood pressure at time of diagnosis (T0) and after 14 years follow-up (T14)

	T0	T14	T0 vs T14 *p*<
SFK with BP > 90%thile	12/55 (22%)	42/55 (76.4%)	0.0001
aSFK	5/17 (29%)	14/17 (82.4%)	0.005
cSFK	7/38 (18%)	28/38 (73.7%)	0.0001
PreHyp-SFK	6/55 (10.9%)	22/55 (40%)	0.0008
PreHyp-aSFK	3/17 (17.6%)	5/17 (29.4%)	NS
PreHyp-cSFK	3/38 (7.9%)	17/38 (44.7%)	0.0005
Hyp-SFK	6/55 (11%)	20/55 (36.4%)	0.003
Hyp-aSFK	2/17 (12%)	9/17 (52.9%)	0.025
Hyp-cSFK	4/38 (10%)	11/38 (29%)	NS

*SFK* all solitary functioning kidney patients; *aSFK* acquired type; *cSFK* congenital type

PreHyp-aSFK: acquired solitary functioning kidney patients with prehypertension

PreHyp-cSFK: congenital solitary functioning kidney patients with prehypertension

Hyp-aSFK: acquired solitary functioning kidney patients with hypertension

Hyp-cSFK: congenital solitary functioning kidney patients with hypertension

At T0, blood pressure was above the 90th centile in 12 children (22%), equally divided between prehypertensive (11%) and hypertensive (11%). At T14 (end of the study period) the number of children with blood pressure >90th centile had increased to 42 (76.4%) (T0 vs T14 *p* <0.0001); which 22 (40%) of them were prehypertensive and 20 (36.4%) were hypertensive (Table 2). Particularly, there was a significant increase of prehypertension (PreHyp) in the cSFK group (T0 3/38 vs T14 17/38 *p* <0.0005), as well as a significant increase of hypertension (Hyp) in the aSFK group (T0 2/17 vs T14 9/17 *p* <0.025) (Table 2).

In particular, subjects with renal scars and hypertension where distributed as follows:

- Acquired solitary functioning kidney, 1/17 at T0 and 2/17 at T14 (i.e., 1/2 hypertensive at T0 and 2/9 at T14)
- Congenital solitary functioning kidney, 2/38 at T0 and 9/38 at T14 *p* < 0.047 (i.e., 2/4 hypertensive at T0 and 9/11 at T14)

In our study we did not detect any subjects with masked hypertension or white coat hypertension.

## Discussion

Current study showed that during a 168-month follow up of children with congenital or acquired solitary functioning kidney, renal functions did not change significantly and blood pressure increased up to over the 90th centile in 76.4% of the studied population. These findings are in agreement with the results of Siomou et al. [4], but in contrast with other reports [5, 6] outlining an alteration of the renal function and/or normal blood pressure values. In our study, children with high blood pressure have apparently normal renal function. What we found, may also be due to the fact that, as described in the literature, nearly 50% of SFK patients have a reduced renal functional reserve [20]: this might help to explain why it is possible to observe the development of high blood pressure without reduction in glomerular filtration rate or increase of serum creatinine levels.

However the higher incidence of subjects with hypertension as shown in our study, albeit higher, is in agreement with those of the KIMONO study (21%) [21, 22]. We believe that the higher hypertension percentage we found, in our study, could be due to the use of ABPM method of measurement, actually representing the most reliable technic of measurement of blood pressure in children [5, 14]. In our study, contrary to other authors [23], we did not detect any cases of masked hypertension. However, we detected a higher number of hypertensive subjects, possibly because of the longer period of follow-up. We suspect that a number of children developed masked hypertension first and by the time they reached the age when we could use ABPM they had progressed to hypertension.

We measured the blood pressure with ABPM at T0 and T14 whenever feasible, i.e., when the child height is ≥120 cm. This was always the case with SFK patients acquired type, but SFK patients congenital type where first observed at a very young age, and ABPM could not be used at T0. It was therefore necessary to compare the IV Report classification [15] with the updated recommendations for the standard assessment of ABPM in children and adolescents [17].). We should observe that data offered by office blood pressure alone do not diverge from the integration of office blood pressure and ABPM. We still support the use of ABPM as it detects white coat hypertension and masked hypertension, and it is the best available tool to assess the effects of antihypertension therapy in the 24 h time span. Nevertheless, the peculiarity of our results is in the classification of the blood pressure status of the subjects who develop values higher than the 90th centile. Up to 36.40% of patients developed hypertension and up to 40% prehypertension; while the former was mostly observed in the acquired SFK group and the latter in the congenital SFK.

Our data differs from a later report by Westland et al. [23] showing a 26% incidence of hypertension and 11% pre-hypertension in children with SFK with a non-statistical significant different distribution between acquired and congenital types of SFK.

Furthermore, our findings have distinct characteristics compared to the reports by Seeman et al. [7]. This study reported hypertension only in cSFK that developed renal scars. On the contrary our finding support hypertension to be more frequently found in association with aSFK; a group of patients who developed a lesser number of renal scars. This observation seems in accordance with a report by Jaoudé et al. suggesting that compensation of renal metabolism by the residual kidney may be more difficult in cases of nephrectomy following trauma [24] and this could account for the higher development of hypertension. We should not underestimate the influence of renal scars on the onset of hypertension. In fact, contrary to acquired solitary functioning kidneys, in the congenital group the number of hypertensive subjects with renal scars increases significantly from T0 to T14. It is worth noting that children with vesicoureteral reflux were excluded from this study. Also of interest, Maheen et al. [25] observed that 21% of otherwise healthy children with newly diagnosed hypertension had renal scars. Seeman et al. [7] recommend a close follow-up for hypertension for all children with renal scars in SFK.

Current study also highlights the presence of prehypertension in 40% of the cases; a condition that we believe requires a close observation. Since prehypertension state is prone to develop into hypertension [26, 27], its early detection in SFK children should be taken into consideration. Therefore adequate renal protective treatment and blood pressure control in these patients should be promptly instituted [28]. Moreover, the increase in blood pressure should be also treated with an increase of sport activity; since it tends to improve the cardiorespiratory fitness in children with SFK [29] and kidney transplantation [30].

## Conclusions

In view of the significant increase in blood pressure over a 14 year follow-up, we suggest prolonged mandatory follow-up of blood pressure every 6 months in children with both acquired and congenital SFK; in view of the higher incidence of prehypertension and hypertension that we found in this population, we consider it more appropriate than the yearly control suggested by Westland et al. [23]. The screening for hypertension should be conducted by clinic blood pressure as well as by ABPM.

Specific attention should be given to the screening of patients for suspect prehypertension; as these children could definitely benefit from simple changes in life style and the early institution of an adequate medical therapy, that effectively prevent the clinical consequences and contrast the progression to hypertension [8].

## Abbreviations

ABPM: Ambulatory blood pressure monitoring
aSFK: Acquired solitary functioning kidney
CAKUT: Congenital anomalies of the kidney and urinary tract
cSFK: Congenital solitary functioning kidney
ESRD: End stage renal disease
GFR: Glomerular filtration rate
Hyp: Hypertension
PreHyp: Prehypertension
SFK: Solitary functioning kidney
